# The Rules of Aseptic and Antiseptic Surgery

**Published:** 1888-04

**Authors:** 


### Book Reviews.

---

THE RULES OF ASEPTIC AND ANTISEPTIC SURGERY: A PRACTICAL TREATISE FOR THE USE OF STUDENTS AND THE GENERAL PRACTITIONER. By Arpad G. Gerster, M. D., Professor of Surgery at the New York Polyclinic, etc. New York. D. Appleton & Co. 1888.

Since Lister first enunciated the great principles of the antiseptic treatment of wounds, there have been published so many conflicting opinions as to the best methods of carrying out these principles, and so many misconceptions of the principles themselves, that we cannot wonder at the bewilderment of the average medical mind at the mere mention of antiseptic surgery. The principle is often lost sight of in vainly seeking to carry out the seemingly useless and innumerable details of its application. As a result there remains in the mind a misty impression that antiseptic surgery consists in buckets of carbolic acid or bichloride solution, numerous solutions for instruments and hands, stacks of vile-smelling gauze and an omnipresent odor of iodoform. These are all used abundantly, and when the wound suppurates, the germ theory is pronounced a fraud and antiseptic surgery a failure.

Dr. Gerster's book will do more towards putting this subject on the basis of common sense and simplicity than anything heretofore published. It will surely dissipate the wide-spread impression that antiseptic surgery will do well enough for large city hospitals, but cannot be practiced anywhere else. The author announces in his preface what will be to many a somewhat startling statement, viz.: "It cannot now be successfully denied that *the surgeon's acts determine the fate of a fresh wound, and*

*that its infection and suppuration are due to his technical faults of omission or commisson."*

If this statement is true (and there are many who believe it is), the surgeon is guilty of gross negligence if suppuration and infection occur in fresh wounds.

Dr. Gerster's work is not a controversial one. He accepts the principles underlying antiseptic surgery as already sufficiently proven. Taking this position as a starting point, he first makes plain the general method of antiseptic treatment and the application of this to special operations. Sepsis and asepsis are defined in the opening chapter. Then follows a description of aseptic wounds and their treatment. He is careful to divest the subject of all mystery, and explains that asepsis and *perfect* cleanliness are identical; as, however, it is next to impossible to secure absolute cleanliness in every particular, it is perfectly rational to employ *chemical sterilization*—that is, some chemical solution which is itself non-irritant to the tissues, but possesses the power of neutralizing any poisonous particles which gain access to the wound. He gives an exceedingly instructive chapter on the "rules of surgical cleanliness," in which are detailed minute directions for preparing sponges, ligatures, sutures, drainage-tubes, disinfecting solutions, and all the different forms of dressings. The practical value of this chapter will be keenly appreciated by every physician whose patients cannot pay for expensive dressings, etc. Dr. Gerster narrates cases illustrating how the antiseptic method may be successfully applied in the most wretched and squalid tenements.

A great deal of space is devoted to the description of special operations, such as amputations, ligature of arteries, extirpation of tumors, operations on the bones, laparotomy, herniotomy and operations on the rectum and urethra.

In describing these operations the special application of the antiseptic method in each case is clearly and concisely given.

In Part II. the etiology and pathology of suppurative processes are considered. Dr. Gerster believes that "the most common source of suppuration is the implantation and thriving in the living human tissues of a minute globular fungus, or micrococcus, called the *staphylococcus pyogenes aureus.*" Accepting this, the treatment of suppuration must naturally be germicidal. The treatment of phlegmon in the various structures of the body according to the antiseptic method is next dealt with in a masterly manner. The antiseptic treatment of tuberculosis, of gonorrhœa and of the external lesions of syphilis occupies the rest of the book.

Throughout the work numerous cases are narrated illustrative of the methods advocated.

Dr. Gerster has given to the profession a work of inestimable value, representing with perfect accuracy the most advanced and scientific pathology and treatment. No physician, be he general practitioner or specialist, can fail to be both interested and instructed by a perusal of this work. The author's style has a crispness and incisiveness that is captivating in the extreme.

The illustrations are mainly from photographs taken by the "flash-light" method and are wonderfully accurate.

The book is printed admirably, and from a typographical standpoint is a great credit to the publishers. H. P. C.

